# Long-term effects of comprehensive school health on health-related knowledge, attitudes, self-efficacy, health behaviours and weight status of adolescents

**DOI:** 10.1186/s12889-018-5427-4

**Published:** 2018-04-18

**Authors:** Nicole Naadu Ofosu, John Paul Ekwaru, Kerry Ann Bastian, Sarah A. Loehr, Kate Storey, John C. Spence, Paul J. Veugelers

**Affiliations:** 1 School of Public Health, University of Alberta, 3-50 University Terrace, 8303 - 112 Street, Edmonton, AB T6G 2T4 Canada; 2 Faculty of Kinesiology, Sport, and Recreation, University of Alberta, 3-113 University Hall, Van Vliet Complex, Edmonton, AB T6G 2H9 Canada

**Keywords:** Comprehensive school health, PA, Healthy eating, Obesity, Health promotion, Public health

## Abstract

**Background:**

APPLE Schools is a Comprehensive School Health (CSH) project, started in schools in socioeconomically disadvantaged areas where dietary habits are poor, physical activity (PA) levels are low, and obesity rates are high. Earlier research showed program effects whereby energy intake, PA and weight status of students in APPLE Schools had reached similar levels as that of students in other schools. However, it is unknown whether the effects of CSH are sustained when children grow into adolescents. Effects of APPLE Schools on health-related knowledge, attitudes, self-efficacy, diet, PA, and weight status, seven years after the start of the project, when students were in junior high and high school were assessed. We hypothesised that APPLE School graduates and comparison school graduates will remain at similar levels for these indicators.

**Methods:**

In the 2015/16 school year, junior high and high school graduates (grades 7–12) in Northern Alberta, Canada participated in a Youth Health Survey. Participants included graduates from APPLE elementary schools (*n* = 202) and comparison elementary schools (*n* = 338). Health-related knowledge, attitudes, self-efficacy, diet (24-h dietary recall), PA (pedometer step count) and weight status were assessed. Mixed effects regression was employed to assess differences in these outcomes between APPLE School graduates and comparison school graduates. Comparisons between elementary school (2008/09) and junior high/high school (2015/16) of self-efficacy, PA and weight status were also conducted.

**Results:**

APPLE School graduates did not significantly differ from comparison school graduates on any outcomes (i.e. knowledge, attitudes, self-efficacy, diet, PA, and weight status). Additionally, no significant differences existed in the comparisons between 2008/09 and 2015/16.

**Conclusion:**

Our findings of no difference between the APPLE School graduates and comparison school graduates suggest that the effects of APPLE Schools may continue into adolescence or the new school environment may have an equalizing effect on the students. Since lifestyle practices are adopted throughout childhood and adolescence, and the school environment has an important influence on development, an extension of CSH initiatives into junior high/high schools should be considered. This will help to consolidate and support the continuance of healthy lifestyle messages and practices throughout childhood and adolescence.

## Background

The school environment is an important setting for promoting and supporting healthy lifestyles among children and youth [1–3]. Schools provide an opportunity to reach a wide range of children over a considerable amount of time. Therefore, enhancing the school environment to promote and support healthy lifestyles can improve children’s health and well-being [4] as well as academic performance [5].

Comprehensive School Health (CSH) is *“an internationally recognised approach to supporting improvements in students’ educational outcomes while addressing school health in a planned, integrated and holistic way”* [6]. This approach may be referred to in other jurisdictions as health promoting schools, coordinated school health and healthy school communities. All of these approaches have similar underlying concepts, which are based on the World Health Organization’s Ottawa Charter for Health Promotion (1986). CSH uses an inclusive approach to promote health and educational achievement by engaging parents, the community and other stakeholders, along with the use of policies and programs to provide supportive social and physical environments [7]. As a population-based approach to health promotion, CSH has the potential to reduce the risk of negative health outcomes by shifting the distribution of risk factors in a favourable direction [8].

APPLE Schools is a school-based health promotion project that uses the CSH approach to create healthy school communities [9]. Though it began in 2008 in ten elementary schools located in socio-economically disadvantaged neighbourhoods, it currently reaches sixty-three school communities in Northern Alberta [9]. The mission of APPLE Schools is to inspire and empower school communities to lead, choose, and be healthy by recommending and supporting measurable and sustainable changes. APPLE Schools aim to effect change in the school, home and community by promoting healthy eating, physical activity (PA) and good mental health. Each school is provided with dedicated staff time in the form of a school health facilitator trained in nutrition, PA, and community development, who works with students, parents, school staff and community members to develop school action plans specific to the needs of each school [9]. School action plans include, but are not limited to, student-led activities that are designed to make healthy living fun and engaging, such as planting classroom gardens, after-school cooking classes and PA programs [9]. Baseline evaluations in 2008 showed that students in schools selected to be part of APPLE Schools had higher dietary energy intake, lower fruit and vegetable intake, lower PA levels and a higher prevalence of obesity compared to other students in Alberta [10]. Subsequent evaluations have established the effectiveness of APPLE Schools in improving diets, increasing PA and reducing the prevalence of childhood obesity [10–12]. However, the long-term effects of APPLE Schools, as with other CSH programs, have not been documented.

Few studies have conducted follow-up assessments on behaviour maintenance or continued effects of school-based interventions in the long-term, beyond the intervention endpoint or outside the intervention environment [13]. The issues associated with the transition from one school environment to another, including losses to follow-up and difficulty acquiring appropriate sample sizes, make long-term evaluations challenging [13, 14]. However, such evaluations are needed to determine behaviour maintenance and continued effects of intervention programs in improving healthy lifestyle habits in school settings, and to justify investments in such programs. We therefore assessed whether the effects of APPLE Schools on health-related knowledge, attitudes, self-efficacy, diet, PA, and weight status are sustained in junior high and high school students who attended an APPLE School in elementary school. Considering the relatively disadvantaged position of APPLE School students at baseline, we hypothesised that junior high and high school students who attended APPLE Schools in elementary school would have knowledge, attitudes, self-efficacy, health behaviours and weight status similar to that of students who did not attend APPLE Schools.

## Methods

### Study population

This research is part of the Return on Investment for Kids’ Health (ROI4Kids) research project, which employs a multidisciplinary approach to evaluate and improve school health programs and policies that promote healthy eating and active living. Students from junior high and high schools in Northern Alberta participated in a Youth Health Survey (YHS) during the 2015/16 school year, seven years after the initial implementation of APPLE Schools in 2008. The sample size for this survey was estimated using a sample size calculator: http://www.sample-size.net/means-sample-sizeclustered/. The estimate was done for PA (pedometer steps per day) taking into account the design effect while using an intraclass correlation coefficient (ICC) of 0.018 [12], a power of 80%, a difference between the APPLE Schools program students and comparison students of 1000 pedometer-measured steps per day, at a 0.05 significance level with adjustment for the expected response rate. Our calculations indicated that a sample size of 403 students would adequately power the study. Additionally, using conservative response and completion rates at the elementary school level of about 40%, and considering incomplete surveys, response and completion rate at the junior high and high school level, we estimated the completion rates to be around 35%, for which we needed to invite 1151 students to participate in the study.

Data for APPLE Schools were first collected in 2008 and 2009. Initial socio-demographic information, knowledge, attitudes, self-efficacy, diet and weight status variables were collected in 2008 while pedometer data were first collected in 2009. Data for the comparison schools were derived from the Raising healthy Eating and Active Living Kids in Alberta (Real Kids Alberta) survey conducted in 2008 and 2009. This is a large population-based survey that collects data on health, nutrition, PA, lifestyle factors, and measured height and weight among grade five students, and data on the school and home environment among their parents and school administrators [12]. The same variables measured in the APPLE Schools evaluation were measured in Real Kids Alberta.

The Human Research Ethics Board and the Cooperative Activities Program of the University of Alberta approved this study, including data collection and parent informed consent.

### Data collection and measures

**Fig. 1:**
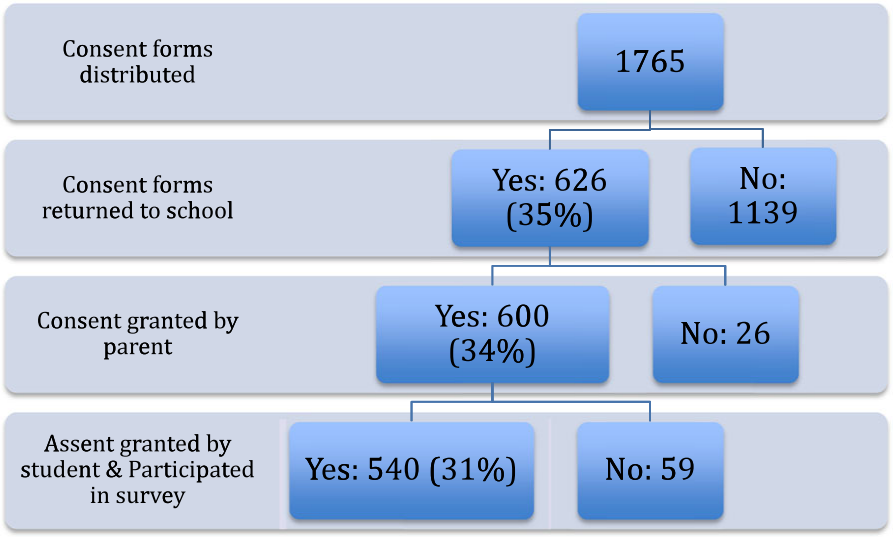
Description of enrolment of Youth Health Survey participants

Trained research assistants collected data in the schools in the 2015/16 school year. School boards were contacted to identify junior high and high schools with a high enrolment of students from APPLE elementary schools. These schools were then invited to participate in the YHS, which comprised of a home survey to be completed by parents at home, and a student survey on knowledge, attitudes, self-efficacy and diet, and an objective assessment of students’ PA and weight status which was conducted in the schools. As part of the student survey, respondents were asked to identify the elementary schools they attended. Based on the schools indicated, and the grades in which they attended these schools, participants were classified as having attended an APPLE School (APPLE School graduate) or not (comparison school graduate). The participation rates of the YHS at each stage of the study are presented in Fig. 1. A total of 1765 home surveys and consent forms were distributed to parents. Of the 626 (35%) students who returned completed home surveys to school, 600 (34%) received parental consent to participate in the study. A total of 540 students completed the YHS (completion rate: 31%).

#### Health-related knowledge, attitudes and self-efficacy

The YHS included questions on health-related knowledge, attitudes, and self-efficacy for healthy eating and active living. Knowledge was assessed using six questions: three related to PA and three related to healthy eating. For PA, participants were asked how strongly they agreed that being physically active influences or affects: i) their health, ii) their body weight, and iii) how well they do in school. For healthy eating, participants were asked how strongly they agreed that the type of food they eat influences or affects: i) their health, ii) their body weight, and iii) how well they do in school. Response options on a four-point scale ranged from ‘strongly agree’ to ‘ strongly disagree’.

Eight questions were used to assess attitude. Participants were asked how much they cared about: i) being physically active, ii) eating healthy foods, and iii) getting enough sleep. Response options on a four-point scale ranged from ‘a lot’ to ‘not at all’. Participants were also asked how strongly they agreed that schools should: i) limit the availability of unhealthy foods, ii) ban the serving of unhealthy foods at school, iii) discourage students from bringing unhealthy foods to school, iv) not allow students to bring unhealthy foods to school, and v) promote healthy eating and active living among students. Response options on a four-point scale ranged from ‘strongly agree’ to ‘strongly disagree’.

Self-efficacy was assessed using eight questions: three related to self-efficacy for PA and five related to self-efficacy for healthy eating. For PA, participants were asked how confident they were that they could be physically active on their own time outside of school hours: i) no matter how tired they might be, ii) even if they had a lot of homework, and iii) on most days of the week. For healthy eating, participants were asked how confident they were that they could: i) eat healthy food at school, ii) choose a healthy snack between school and dinner, iii) eat healthy food or choose a healthy snack when with friends, iv) choose a healthy snack when alone at home, and v) choose a healthy snack when bored or sad. Response options on a four-point scale ranged from ‘very confident’ to ‘not at all confident’.

A score (range 1 to 4) was assigned to each response option and confirmatory factor analysis with varimax rotation was used to confirm four factors and to generate factor scores Internal consistency of the scale items for four factors was high (Cronbach’s α for: knowledge = 0.80; attitude = 0.75; self-efficacy for healthy eating = 0.83; self-efficacy for PA = 0.77). For each factor (knowledge, attitudes, self-efficacy for PA, and self-efficacy for healthy eating), responses were summed across all items to obtain an easy to interpret composite score, which was used to characterize graduates from APPLE Schools and comparison school graduates for the purpose of descriptive analyses [15]. However, factor scores were used for the statistical models.

#### Dietary intake

The students completed an online 24-h dietary recall using the Waterloo Eating Behaviour Questionnaire (WEB-Q 24), which has been validated for use with children and youth [16–19]. Participants’ caloric intake was calculated based on recorded intake from the online 24-h dietary recall and from the Canadian Nutrient File [20].

#### Pedometer-determined physical activity

PA was measured in the form of hourly step counts recorded over nine consecutive days, using the Omron HJ-720 ITC time-stamped pedometer (Omron Canada Inc., Toronto, Ontario, Canada). The validity of the Omron time-stamped pedometer has been demonstrated under various conditions [21, 22]. Trained research assistants explained to students how to use the pedometers. Students were asked to wear the pedometers on the right hip directly in line with the knee during all waking hours except when showering, swimming, or participating in any activities that an adult regarded as unsafe to wear the pedometer. Students had the opportunity to receive daily text message reminders to wear their pedometers. Because of variations in administration and collection of the pedometers in each school, step count records from the first and ninth day were excluded from the analyses. Pedometer readings were considered complete if the pedometer was worn for a minimum of eight consecutive hours per day on at least two school days and one non-school day (weekend and/or holiday).

Steps during school hours (8:00 am – 3:59 pm) and non-school hours (7:00 am – 7:59 am and 4:00 pm – 8:59 pm) were considered for the PA assessment. Steps were normalised to hourly-accumulated steps during these periods by dividing total steps during school hours and non-school hours by eight and six hours, respectively. Average steps during school days and non-school days steps were also estimated. Students’ step counts were averaged to represent a typical week (i.e. five school days and two non-school days).

#### Weight status

Student standing height was measured using a Seca 213 stadiometer (Seca gmbh & co., Hamburg, Germany) to the nearest 0.1 cm after students had removed their shoes. Body weight was measured to the nearest 0.1 kg on a calibrated digital scale (Health o Meter®, Sunbeam Products, Inc., USA). Body mass index (BMI) was calculated as weight divided by height squared (kg/m^2^). Obesity and overweight were defined using age and sex specific categories of the World Health Organization standard for children and youth [23].

#### Socio-demographic information

Students’ gender and age were self-reported. Information on geographic residence (metropolitan, city, rural-town), household income (<$50,000, $50,001–$100,000, and > $100,000) and parental education attainment (secondary or less, college, university or above) were reported by parents and used as a proxy for socioeconomic status.

### Statistical analyses

All analyses were conducted using the STATA version 14 software [24]. Differences between APPLE School and comparison school graduates were assessed using the Chi-square test or *t*-test where appropriate. As observations of students are nested within those of their schools, mixed effects regression models were employed to examine differences between APPLE School graduates and comparison school graduates. Unstandardized regression coefficients and 95% confidence intervals (CI) were obtained from the multivariable cross-sectional comparisons of APPLE School graduates and comparison school graduates (2015/16) for the outcomes knowledge, attitude, self-efficacy (for healthy eating and for PA), diet (dietary energy intake) and PA (step counts for typical week, school days, non-school days, school hours, and non-school hours). Odds ratios (OR) and 95% CI were obtained for weight status outcomes (overweight and obesity).

Cross-sectional comparisons of the assessments of self-efficacy, PA and weight status in elementary school (2008/09) and junior high/high school (2015/16) were also examined. Only these variables were used as they had comparable measures in 2008/09. The multilevel regression analyses were adjusted for the confounding potential of gender, age, geographic residency, household income, and parental education. An interaction term, defined as the product of the year variable (2008/09 = 0, 2015/16 = 1) and the binary intervention variable (Comparison Schools = 0, APPLE Schools =1), was included in the multilevel models to estimate the difference in regression coefficients and OR, for the outcomes as a measure of intervention effect, i.e. the difference between APPLE School students and graduates relative to the difference between comparison school students and graduates.

## Results

The characteristics of students are shown in Table 1. Our sample included 13 junior high/high schools with an average of 41 participants from each school. The comparison school graduates included more girls (59.1% vs. 48.3%; *p* = 0.021) and had a higher mean age (14.0 years vs. 13.8 years; *p* = 0.045) than the APPLE School graduates. Significant differences existed in household income and geographic location (*p* < 0.001), with greater proportions of APPLE School graduates being from families earning more than $100,000 per year and residing in cities. APPLE School graduates also reported lower health-related attitudes (2.72 vs. 2.81; *p* = 0.04) and a higher percentage of overweight (44.6% vs. 32.3%; *p* = 0.005) in comparison with graduates from comparison schools. No statistically significant differences were found for knowledge, self-efficacy, diet, PA-step counts, and obesity.

**Table 1:** Characteristics of APPLE School students, APPLE School graduates, comparison schools students and comparison schools graduates

	2008/09		2015/16		*p* ^*^
	APPLE Schools	Comparison Schools	APPLE Schools graduates^***^	Comparison Schools graduates^***^	
No. of schools	10	163	13	13	
No. of students	277	3300	202	338	
Gender, %					
Girls	50.2	52.0	48.3	59.1	0.021
Boys	49.8	48.0	51.7	40.9	
Age, mean ± SD (years)	10.8 ± 0.4	10.9 ± 0.4	13.8 ± 1.4	14.0 ± 1.3	0.045
Knowledge (mean ± SD)^**^	2.73 ± 0.69	2.81 ± 0.71	3.26 ± 0.54	3.34 ± 0.49	0.07
Attitude (mean ± SD)^**^	3.42 ± 0.55	3.44 ± 0.57	2.72 ± 0.51	2.81 ± 0.41	0.04
Self-efficacy for healthy eating (mean ± SD)^**^	3.12 ± 0.63	3.11 ± 0.61	2.77 ± 0.73	2.81 ± 0.65	0.51
Self-efficacy for physical activity (mean ± SD)^**^	2.99 ± 0.63	3.09 ± 0.59	2.91 ± 0.76	2.83 ± 0.71	0.259
Dietary outcomes					
Mean dietary energy intake (kcal)/d ± SD	2117 ± 1242	1998 ± 1157	2173 ± 1034	2155 ± 1059	0.848
PA, mean ± SD					
Typical week, steps/d	9081 ± 2638	9798 ± 2960	6810 ± 2549	6667 ± 2586	0.615
School days, steps/d	9943 ± 2834	10,540 ± 3242	7616 ± 2833	7413 ± 2960	0.528
Non-school days, steps/d	6928 ± 3799	7944 ± 3851	5177 ± 3476	5067 ± 3188	0.787
School hours, steps/h	777 ± 218	839 ± 245	653 ± 221	634 ± 231	0.445
Non-school hours, steps/h	621 ± 300	638 ± 55	340 ± 222	323 ± 227	0.488
Weight status					
Overweight, %	44.4	37.6	44.6	32.3	0.005
Obesity, %	19.5	14.0	18.7	15.7	0.381
Parental education, %					
Secondary or less	30.5	27.2	23.0	21.8	0.112
College	41.1	42.1	32.8	24.9	
University or above	28.5	30.7	44.3	53.2	
Household income, %					
< $50,000	34.5	21.6	13.7	24.8	*p* < 0.001
$50,001 - $100,000	37.4	40.4	15.3	34.2	
> $100,000	28.1	38.0	71.0	41.1	
Geographic Location, %					
Metropolitan	64.9	24.9	23.8	60.2	*p* < 0.001
City	0.0	30.8	76.2	39.8	
Rural-town	35.1	44.3	–	–	

^*^p < 0.05 – statistically significant difference between APPLE School graduates and comparison school graduates

^**^Mean score on the four-point scale (please see text)

^***^Average number of participants from each school was 41

Table 2 shows a cross-sectional comparison of graduates of APPLE Schools and comparison schools. APPLE School graduates did not significantly differ from comparison school graduates with respect to health-related knowledge, attitude, self-efficacy, diet, PA and weight status.

**Table 2:** Cross-sectional comparison of APPLE School graduates and comparison school graduates on knowledge, attitudes, self-efficacy, diet, physical activity and weight status

Variable	(coefficient and 95% CI)^a^
Knowledge^b^	−0.15 (− 0.39, 0.09)
Attitude^b^	− 0.16 (− 0.42, 0.09)
Self-efficacy for healthy eating^b^	− 0.15 (− 0.39, 0.08)
Self-efficacy for physical activity^b^	0.14 (− 0.10, 0.39)
Dietary outcomes	
Dietary energy intake (kcal/d)	−75.88 (− 316.65, 164.89)
Physical activity	
Typical week, steps/d	−149 (− 865, 567)
School days, steps/d	−303 (− 1113, 508)
Non-school days, steps/d	−76 (−1177, 1026)
School hours, steps/h	−9 (−66, 48)
Non-school hours, steps/h	−16 (−79, 48)
Weight status	(odds ratio and 95% CI)^a^
Overweight	1.25 (0.76, 2.08)
Obesity	0.99 (0.53, 1.85)

^a^Model adjusted for gender, age, parental educational attainment, household income and geographic location

^b^Used factor scores from the confirmatory factor analyses

Negative values of β and OR values below 1 indicate lower values among APPLE schools graduates relative to comparison schools

Comparisons between elementary school (2008/09) and junior high/high school (2015/16) of self-efficacy, PA and weight status are presented in Table 3. After adjusting for covariates, the analysis showed that between 2008/09 and 2015/16, no statistically significant differences existed in self-efficacy for PA and self-efficacy for healthy eating. PA declined between 2008/09 and 2015/16 for both APPLE School graduates and comparison school graduates. The difference in step count between 2008/09 and 2015/16 in APPLE School graduates was not statistically different from the observed difference in comparison school graduates. The comparison of weight status between elementary school (2008/09) and junior high/high school (2015/16), also showed no statistically significant differences between the two groups.

**Table 3:** Comparisons of self-efficacy, PA and weight status between elementary school (2008/09) and junior high/high school (2015/16)

	APPLE Schools^a^^†^ (Difference between graduates, 2015/16 and students, 2008/09)	Comparison schools^a^^†^ (Difference between graduates, 2015/16 and students, 2008/09)	Difference between graduates, 2015/16 and students, 2008/09 in APPLE Schools relative to comparison schools^a^^‡^ (group x time interaction)	ICC
Self-efficacy (coefficient and 95% CI)^b^				
Self-efficacy for healthy eating (β and 95% CI)	0.14 (−0.22,0.49)	0.30 (− 0.02, 0.62)	− 0.16 (− 0.47, 0.14)	0.048
Self-efficacy for PA (β and 95% CI)	0.18 (− 0.010, 0.47)	−0.001 (− 0.27, 0.27)	0.19 (− 0.09, 0.45)	0.007
PA (coefficient and 95% CI)				
Typical week, steps/d	− 776 (− 2171, 620)	− 1571 (− 2912, − 230)	795 (− 317, 1908)	0.085
School days, steps/d	− 608 (− 2160, 944)	− 1260 (− 2747, 227)	652 (− 582, 1886)	0.096
Non-school days, steps/d	− 1150 (− 2985, 686)	− 1882 (− 3638, − 125)	732 (− 770, 2235)	0.042
School hours, steps/h	54 (−76, 184)	−32 (−154, 90)	86 (−17, 186)	0.182
Non-school hours, steps/h	−235 (− 369, −102)	− 244 (− 374, −114)	8 (−101, 117)	0.041
Weight status (odds ratio and 95% CI)				
Overweight	0.82 (0.44, 1.54)	0.85 (0.46, 1.56)	0.96 (0.54, 1.72)	0.017
Obesity	1.35 (0.59, 3.12)	2.36 (1.07, 5.20)	0.57 (0.27, 1.20)	0.039

^a^Model adjusted for gender, age, parental educational attainment, household income, and geographic location

^b^Used factor scores from the confirmatory factor analyses

^†^Negative values of β and OR values below 1 indicate a lower value of the outcome in graduates relative to students

^‡^Negative values of β and OR values below 1 indicate a lower value of the outcome in APPLE Schools relative to comparison schools between 2008/09 and 2015/16

## Discussion

We assessed whether the effects of APPLE Schools are sustained in junior high and high school students who attended elementary schools participating in the project. APPLE School graduates did not significantly differ from comparison school graduates with respect to all outcomes (i.e. health-related knowledge, attitude, self-efficacy, diet, PA, and weight status. Comparisons of self-efficacy, PA and weight status in elementary school (2008/09) and junior high/high school (2015/16), also showed no statistically significant differences between the two groups.

APPLE School students started worse off with regards to healthy dietary habits, PA levels and obesity prevalence relative to other students [10]. However, within two years of the APPLE Schools program, they showed substantial improvements such that energy intake, PA and weight status of students had become similar as that of students in comparison schools [10]. We had hypothesised that the effects of the APPLE Schools program would remain and thus the absence of a difference between students from APPLE Schools and comparison schools would continue into junior high/high school. Therefore, finding no significant differences between the two groups suggests a possibility that the effects of APPLE Schools continue into junior high/high school. However, since both groups are now in the same junior high/high school environment, the lack of significant difference between the two groups could also be because the new school environment has an equalizing effect on the students regardless of where they started.

The decrease in PA-step counts between elementary school and junior high/high school in both APPLE School graduates and comparison school graduates reflects observations from other studies that PA generally declines in the transition from childhood through to adulthood [13, 25–28]. This decline likely reflects the biological processes related to growth and maturation [29, 30], and possibly the increasing social demands at the different life stages [28]. Since lifestyle practices and habits are developed both in childhood and adolescence [31, 32], the school environment can play an important role in promoting and supporting healthy lifestyles among children and youth [1–3]. As the junior high/high school environment also exerts its own influence on student behaviours [33–35], it is therefore insufficient to focus successful CSH programs only on elementary schools. Thus, there is a reasonable expectation that extending CSH programs into junior/high schools could mitigate the reduction in PA during adolescence, and consolidate healthy lifestyle messages and practices adopted in elementary school.

Some studies have assessed long-term effects of school-based health promotion, most of which are focused on PA outcomes. Lai, et al. [13] systematically reviewed school-based interventions that focus on PA to assess whether they produced a sustained impact in children and adolescents. ‘Follow-up assessment’ was defined as data collection at least six months after post-intervention testing. Of the fourteen studies identified, ten studies measured and reported a sustained impact in PA. However, some reported a sustained impact only for boys or only for girls, and nine studies used self-reported methods of assessment. Tarro, et al. [14] and Nader, et al. [36] also reported sustained effects on PA, two and three years respectively after the cessation of the school-based intervention. These findings too were based on self-reported PA. Tarro, et al. [14] also reported a reduced obesity prevalence. In contrast, Meyer, et al. [37], objectively measured PA, three years after the cessation of an intervention in elementary schools. They found that apart from aerobic fitness, previously observed beneficial effects on PA (accelerometer measurements) and body fat after one year were not sustained in the intervention arm. The relatively short duration of the intervention (nine months) may have impacted the sustainability of the intervention. Systematic reviews of school-based PA programs among children and adolescents show that duration, frequency and intensity of interventions can influence the effectiveness of the interventions [13, 38]. Thus, the most effective programs have characteristics such as being of long duration and high intensity, involving the whole school, being a multifactorial intervention, and comprising changes to the school environment [39]. These are characteristics of APPLE Schools as well as some other CSH-oriented programs, which have demonstrated beneficial effects on students’ diet, PA and weight status [35, 39, 40].

To our knowledge, this is the first study aimed at assessing the long-term effects of CSH (7 years after the initial implementation) on multiple outcomes – health-related knowledge, attitude, self-efficacy, diet, PA, and weight status. The strengths of this study include the uniqueness of the APPLE Schools project and the use of objective measures for PA and weight status. This study is not without limitations. First, we were unable to separate the effects of APPLE Schools from the effects of the new school environment because of the study design. High school students are likely to have spent a shorter time in APPLE Schools compared with junior high school students. However, our sample sizes were inadequate for the separate analyses, and such analyses may raise concerns about biases in attributing differences to an eroded effect of APPLE Schools. Furthermore, we did not account for duration (i.e. how long the elementary school had been an APPLE School) and intensity of the APPLE Schools intervention (i.e. number of days per week that the school had access to a school health facilitator). Varying durations and intensities in APPLE Schools could have impacted the outcomes of interest measured. The use of one 24-h recall instead of repeated 24-h recalls allowed the assessment of average intake at a group level but not the usual intake at an individual level. Another limitation is the cross-sectional design, by which causality cannot be established. Incomes in the more northern areas of Alberta, are inflated because of the economic boom and labour demands, which do not reflect on levels of education. Thus, parental education may be a better proxy for socioeconomic status in this sample rather than income. However, we adjusted for the socioeconomic status proxies (parent education, household income, geographic residence) in our analyses.

## Conclusion

Our findings of no difference between APPLE School graduates and comparison school graduates suggest that either the effects of CSH still continue into adolescence or the new school environment may have an equalizing effect on the students regardless of where they started. However, since lifestyle practices are adopted throughout childhood and adolescence, and the school environment is recognised as an important influence on children and adolescents’ development, an extension of CSH initiatives into junior high/high schools should be considered. This will help to consolidate and support the continuance of healthy lifestyle messages and practices throughout childhood and adolescence.

## Abbreviations

APPLE Schools: A Project Promoting healthy Living for Everyone in Schools
CI: Confidence interval
CSH: Comprehensive school health
ICC: Intraclass Correlation Coefficient
OR: Odds ratio
PA: Physical activity
ROI4Kids: Return on investment for kids’ health
YHS: Youth health survey
